# Identifying Demographic and Clinical Determinants of Ischemic Workup in Patients with Heart Failure

**DOI:** 10.3390/jcm13237279

**Published:** 2024-11-29

**Authors:** Kristen M. John, Peter Wenn, Ofek Hai, Roman Zeltser, Amgad N. Makaryus

**Affiliations:** 1 Department of Cardiology, Donald and Barbara Zucker School of Medicine at Hofstra/Northwell, Hempstead, NY 11549, USA; 2 Division of Cardiology, HCA Brandon Regional Hospital, Brandon, FL 33511, USA; 3 Department of Cardiology, Nassau University Medical Center, East Meadow, NY 11554, USA

**Keywords:** heart failure, coronary artery disease, ischemic

## Abstract

**Background/Objectives:** Coronary artery disease (CAD) is a common and treatable cause of heart failure (HF), but ischemic evaluation is often overlooked when evaluating patients with new-onset HF. Here, we sought to discern demographic and clinical predictors of ischemic workup in patients with newly diagnosed HF. **Methods:** A retrospective study of 200 consecutive patients with new-onset HF admitted to our safety-net hospital between 2011 and 2015 was performed. We developed a multivariate logistic regression model to analyze determinants of undergoing ischemic evaluation. **Results:** A total of 99 patients (49.5%) underwent ischemic workup, while 101 patients (50.5%) did not. The mean age of the cohort was 73.9 ± 16, with 50% as male and 51% as White. In total, 41.5% of patients had HF with reduced ejection fraction, and 37% of patients had HF with preserved ejection fraction. Among the patients who underwent ischemic evaluation, 63.6% received nuclear stress testing, 24.2% received cardiac catheterization, 9.1% received stress echocardiography, and 3% received computed tomography angiography. Demographic and clinical factors such as sex, age, race, presence of hypertension, hyperlipidemia, chronic kidney disease, diabetes, or obesity had no significant association with receiving ischemic workup (*p* > 0.05). Patients with known CAD (OR 2.816, *p* = 0.015) and a higher social deprivation index (SDI) (OR 1.022, *p* = 0.003) were significantly more likely to receive an ischemic evaluation. Atrial fibrillation was significantly negatively associated with receiving ischemic workup (OR: 0.24; *p* = 0.001). **Conclusions:** In our single-center safety-net hospital analysis, known CAD and higher SDI were significant predictors of ischemic evaluation in patients with newly diagnosed HF. Multiple demographic features, including age, sex, race, and clinical features, including HF type, hypertension, hyperlipidemia, and diabetes, had no significant correlation with ischemic workup.

## 1. Introduction

Heart failure bears a high burden of mortality, contributing to over 10% of deaths annually in America [1]. Over one million patients are diagnosed with heart failure each year in the United States, and approximately two-thirds of heart failure cases can be attributed to pre-existing CAD [2,3,4]. Ischemic evaluation is often minimally invasive or non-invasive, including modalities such as exercise stress testing, echocardiography, and diagnostic coronary angiography. Nonetheless, testing is often underutilized in patients with newly diagnosed heart failure.

Studies have shown that the majority of patients presenting with new-onset heart failure do not receive any ischemic workup [5,6]. One study, using data from the MarketScan Commercial and Medicare Supplemental databases, found that, of patients with new-onset HF diagnosed during an inpatient hospitalization, only 17.5% of patients underwent CAD testing during the admission, increasing to 27.4% within the next 90 days with a revascularization rate of 4.3% [5]. During the early stages of disease progression, CAD patients may only present with minor symptoms or remain asymptomatic, making it unlikely to prompt further ischemic evaluation. Notably, asymptomatic CAD is highly prevalent among patients with type 2 diabetes mellitus [7]. It was also shown that hypertension, hyperlipidemia, HF with reduced ejection fraction, mixed or unspecified HF, and smoking were associated with increased odds of ischemic evaluation. Another study, utilizing the GWTG-HF registry, showed that CAD testing occurred in <40% of patients with newly diagnosed HF and identified younger age, male sex, smoking history, hyperlipidemia, and left ventricular ejection fraction category as factors that increased the likelihood of CAD testing [6]. These results, highlighting the underutilization and variability of ischemic evaluation and revascularization in patients with new-onset HF, are particularly concerning in the context of the STICHES trial, which demonstrated lower rates of all-cause mortality among patients who underwent coronary artery bypass grafting in conjunction with medical therapy compared to medical therapy alone [8]. The findings from the STICHES trial can be attributed to a significant reduction in risk-adjusted mortality with coronary artery bypass grafting, likely due to advances in myocardial protection techniques, surgical techniques, and perioperative care [8].

More recent research has acknowledged geographical, institutional, and physician-based variability in rates of ischemic evaluation, warranting further investigation of these differences to help tailor and enhance strategies to increase rates of testing in HF patients [2,9]. Our study is based on patient health records from a safety net hospital, which reflects a patient population largely from underserved communities. In this regard, this retrospective analysis addresses a patient population often overlooked within the national healthcare system. Our aim was to elucidate the clinical and demographic factors that are predictive of ischemic workup in newly diagnosed patients with heart failure at our tertiary care center.

## 2. Materials and Methods

A total of 200 consecutive new-onset heart failure patients admitted to our hospital, Nassau University Hospital, a safety-net tertiary care hospital in New York, between 2011 and 2015 were evaluated. Institutional Review Board approval was obtained for this retrospective cohort study (IRB #21-386).

Demographic features were gathered, including age, sex, race, and social deprivation index (SDI), correlated from zip codes using data from the contemporaneous American Community Service, a project of the US Census Bureau [10]. Race was stratified into White, Black/African American, Asian/Hawaiian/Pacific Islander, and Other. Clinical features were also recorded, including the type of heart failure (HF with reduced ejection fraction, HF with preserved ejection fraction, HF with mid-range ejection fraction, or HF with improved ejection fraction) and diagnoses of coronary artery disease, hypertension, hyperlipidemia, atrial fibrillation, chronic kidney disease, diabetes mellitus, and obesity. Baseline patient demographic and clinical characteristics are described using proportions for categorical variables and means with standard deviations for continuous variables.

A multivariate logistic regression model was constructed to determine characteristics associated with receiving an ischemic evaluation. Ischemic evaluation was defined as any of the following: stress echocardiography, nuclear stress imaging, computed tomography coronary angiography, and cardiac catheterization. The tolerance and variance inflation factors were calculated to evaluate the collinearity of the potential variables. The Hosmer–Lemeshow test was performed to evaluate the goodness-of-fit of the constructed model. All analyses were conducted using SPSS (IBM™ SPSS Statistics for Macintosh, Version 29.01.0, Armonk, NY, USA) using a *p*-value ≤ 0.05 for statistical significance.

## 3. Results

**Figure 1:**
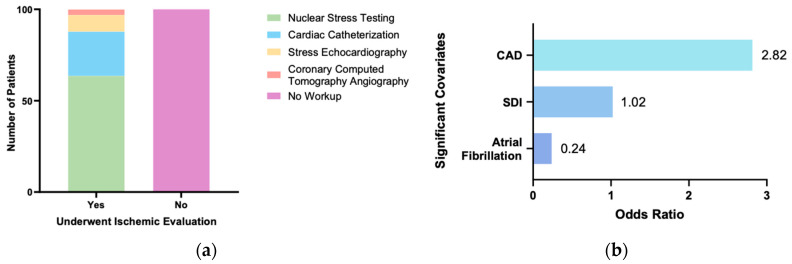
(**a**) Breakdown of ischemic evaluation by modality; (**b**) covariates determined as significantly positively or negatively associated with undergoing ischemic evaluation in our study population.

The baseline demographic and clinical characteristics of the study population are shown in Table 1. The average patient age was 73.9 years old. There were 100 men (50%) and 100 (50%) women included in this study. A total of 99 patients (49.5%) received ischemic evaluation, while 101 patients (50.5%) did not receive ischemic evaluation. Of those who received ischemic evaluation, 63.6% underwent nuclear stress testing, 24.2% underwent cardiac catheterization, 9.1% underwent stress echocardiography, and 3% underwent coronary CT angiography (Figure 1a). From these data, a multivariate logistic regression model was constructed to identify factors associated with ischemic evaluation, which is shown in Table 2. The model was statistically significant (X^2^(16) = 73.879, *p* < 0.001) and correctly classified 75% of cases with a Nagelkerke R^2^ value of 0.44. This Nagelkerke R^2^ value indicates that the model predictors explain approximately 44% of the variance in ischemic evaluation, suggesting that the model is moderately strong and can reliably provide insight into the relationships between the characteristics assessed and whether ischemic evaluation was performed. All variables had variance inflation factors of <2 and tolerances of >0.5, suggesting no significant collinearity among them. The Hosmer–Lemeshow goodness-of-fit evaluation indicated that the model fit the data well, suggesting no significant difference between observed and predicted values (*p* = 0.238). Several characteristics were identified as associated with whether a patient underwent ischemic evaluation (Figure 1b). Notably, patients with known CAD (OR 2.816, *p* = 0.015) were found to be more likely to receive ischemic evaluation. Furthermore, patients with higher SDI, a marker of lower socioeconomic resources often associated with higher cardiovascular morbidity and mortality, were also more likely to undergo ischemic workup (OR 1.022, *p* = 0.003). In contrast, those with atrial fibrillation were less likely to undergo ischemic evaluation (OR: 0.240; *p* = 0.001). Several demographic characteristics, including age, sex, and race, were not significantly associated with whether a patient received ischemic evaluation. Furthermore, although multiple clinical characteristics, including diagnoses of chronic kidney disease, diabetes mellitus, hypertension, hyperlipidemia, and obesity, were associated with an increased likelihood of undergoing ischemic evaluation, these associations were not significant.

## 4. Discussion

Prior studies have suggested that the variability in ischemic testing between geographical regions, health institutions, and physicians should be further studied in order to develop and implement more effective approaches to improve rates of testing in patients with new-onset heart failure [2,9]. Our aim was to determine which clinical and demographic factors were predictive of ischemic workup in newly diagnosed patients with heart failure at our tertiary care center. Through our single-center safety-net hospital analysis, we determined that known CAD and higher SDI had a significant positive association with receiving ischemic workup, while atrial fibrillation had a significant negative association with receiving an ischemic workup.

CAD is a highly prevalent and treatable condition that significantly contributes to heart failure. Prior research has demonstrated that CAD is independently correlated with poorer long-term outcomes for patients with heart failure [11,12]. This correlation underscores the importance of identifying and addressing CAD in this vulnerable patient population. To this end, the 2022 guidelines from the American Heart Association, American College of Cardiology, and Heart Failure Society of America guidelines note that, in patients with heart failure, evaluation for ischemic heart disease can provide utility in determining etiology and management, while, in those with concomitant heart failure and CAD who are eligible for revascularization, noninvasive stress imaging may be considered [13,14]. Despite these recommendations, less than 40% of incident heart failure patients are evaluated for CAD, and rates of CAD testing have remained relatively stagnant over the years [2]. We found that patients with CAD were 2.8 times more likely to have undergone ischemic evaluation than those without CAD, suggesting good concordance with recommended guidelines. Early CAD testing (within 1–3 months of HF diagnosis) can decrease overall mortality rates, primarily through treatment initiation and surgical intervention [8].

We also observed that atrial fibrillation was significantly negatively associated with receiving an ischemic workup. This is possibly due to the attribution of atrial fibrillation-related tachycardia cardiomyopathy as the primary etiology of a patient’s heart failure, thus diminishing the suspicion of concomitant ischemic coronary disease. Given the overlap in symptoms common to atrial fibrillation and ischemic heart disease, it is possible for clinicians to misattribute these symptoms solely to the former without considering the latter. Furthermore, patients presenting with symptomatic atrial fibrillation commonly have elevated troponin levels, which can occur in both atrial fibrillation and ischemic heart disease due to increased myocardial oxygen demand. Studies have suggested that a mild troponin elevation in patients with atrial fibrillation is an unreliable indicator for significant underlying ischemic disease, which may deter further workup, further contributing to lower rates of ischemic evaluation in this patient population [15]. Nonetheless, heart failure and atrial fibrillation have a complex relationship as atrial fibrillation has been thought to accelerate atherosclerosis through systemic inflammation, endothelial dysfunction, and increase myocardial oxygen demands, resulting in the development or exacerbation of ischemic disease, and so ischemic testing in these patients should not be foregone [16].

Ischemic heart disease is the leading cause of HF with reduced ejection fraction in developed countries, with one study reporting CAD as the underlying etiology of over 60% of cases of HF with reduced ejection fraction [17]. While the role of CAD in HF continues to be investigated, research suggests that CAD-induced left ventricular or myocardial dysfunction contributes to both the initial development and progression of HF with reduced ejection fraction [17]. Studies have suggested that patients with HF with reduced ejection fraction and HF with mid-range ejection fraction were more likely to undergo ischemic evaluation. Furthermore, an ischemic evaluation performed in patients with new-onset HF with reduced ejection fraction within 90 days of hospitalization was linked to nearly a 30% decrease in HF readmission and mortality rates [18]. In our model, however, heart failure type was not a significant predictor of testing, though HF with reduced ejection fraction was the most represented type of heart failure in those receiving ischemic evaluation [2,6]. Our findings may suggest a potential gap in care as more HF with reduced ejection fraction patients could benefit from higher rates of ischemic assessment. Furthermore, while diagnoses of other diseases associated with cardiovascular morbidity, such as hypertension, hyperlipidemia, chronic kidney disease, diabetes, and obesity, increased the likelihood that a patient with heart failure underwent ischemic evaluation, these associations were not statistically significant. Data from the Atherosclerosis Risk in Communities show that, while rates of CAD in HF with reduced ejection fraction patients have declined over time, comorbidities associated with HF, such as hypertension and atrial fibrillation, have increased [18]. Therefore, the shifting trends in CAD prevalence and relevant comorbidities in HF patients highlight the need for a continuously evolving approach toward disease management. Given the complex relationship between heart failure and ischemic disease, researchers have recently developed the HLM score, a pathophysiological-based prognostic tool for patients with HF that assesses heart damage, lung involvement, and systemic multiorgan involvement that has demonstrated promising prognostic power in patients with HF due to ischemic heart disease [19,20].

SDI is a composite measure of area level deprivation based on seven demographic characteristics obtained from the American Community Survey, including percent living in poverty, percent with less than 12 years of education, percent single-parent households, percent living in rented housing units, percent living in overcrowded housing units, percent of households without a car, and percent non-employed adults under 65 years old [10]. These characteristics are assessed at multiple geographic levels and are analyzed through factor analysis to identify underlying relationships with factor loadings used to weight each component, including only variables with a factor loading greater than 0.60, to provide a standardized measure of regional social deprivation [9]. This is particularly relevant in our area of study as higher socioeconomic deprivation has been linked to higher cardiovascular morbidity and mortality [21]. More specifically, lower family income and educational background, which are accounted for in the calculation of SDI, were found to be associated with increased rates of cardiac complications in patients newly diagnosed with heart failure [22]. Consequently, patients with lower SDI and newly diagnosed heart failure would likely benefit significantly from undergoing ischemic evaluation, which could guide strategies to prevent or mitigate these cardiac complications. Although patients with higher SDI are often less likely to undergo evidence-based therapy, in our analysis, we found that patients with higher SDI were more likely to undergo ischemic evaluation [23]. This is reasonable considering that our institution is a safety-net hospital with a largely underserved patient population, and these results align with prior studies at our institution that have demonstrated equitable treatment of hospitalized patients with heart failure regardless of socioeconomic status [24].

Several other demographic features, including age, sex, and race, had no significant association with whether a patient received ischemic evaluation in our model. Studies have suggested that elderly patients (>80 years old) were more likely to get tested than middle-aged patients (40–64 years old); women were less likely to get tested than men; and Black and Asian patients were less likely to get tested than White patients [2]. At our institution, based on their odds ratios, women were less likely to undergo ischemic evaluation compared to men, and individuals who identified as Black or African American were more likely to undergo evaluation, while individuals who identified as Asian were less likely to undergo evaluation; however, these relationships were not statistically significant in the regression analysis.

Of the modalities to evaluate for CAD, nuclear stress testing was utilized the most frequently, occurring in over half of all patients who underwent workup, followed by cardiac catheterization, stress echocardiography, and coronary CT angiography. For CAD evaluation, exercise electrocardiogram is generally first-line as it is widely available, inexpensive, does not require peripheral access or radiation exposure, and assesses functional capacity with good sensitivity (68%) and specificity (77%) [25]; its use is contraindicated in those unable to exercise or with specific baseline electrocardiogram abnormalities. Nonetheless, this modality was not applicable to our study population of inpatients admitted for new-onset heart failure. Nuclear stress testing is a form of radionuclide imaging that primarily encompasses two major techniques: single photon emission computed tomography and positron emission tomography. Single photon emission computed tomography is used most often, while positron emission tomography, though it offers superior visual quality, is used less frequently due to high cost and limited availability. Single photon emission computed tomography and positron emission tomography offer similar specificities, though the latter provides greater sensitivity (93%) compared to the former (88%) [26]. Another non-invasive modality for CAD testing is stress echocardiography, which demonstrates higher sensitivity (79%) and specificity (87%) compared to exercise electrocardiogram and higher specificity but lower sensitivity compared to nuclear perfusion imaging [27]. However, stress echocardiography can be prone to poor image quality due to the patient body habitus or lung disease and operator technique. Coronary CT angiography has a high negative predictive value and so is recommended for patients with a low–intermediate risk of CAD. While CCTA is relatively readily available and effective in evaluating atherosclerosis burden and functional significance of stenosis, there are numerous patient-specific characteristics that can reduce image quality. Furthermore, it necessitates the use of iodinated contrast and involves some radiation exposure. Finally, cardiac catheterization is considered the gold standard for the diagnosis of CAD, but it is invasive and costly, so the aforementioned non-invasive strategies are preferentially recommended for initial CAD evaluation. Therefore, it is reasonable that nuclear stress testing was the most utilized modality for ischemic evaluation in our study population, given its availability, high sensitivity, and ability to be performed on virtually any patient.

While our study provides unique insight into some of the clinical and demographic characteristics predictive of ischemic evaluation in patients with heart failure at a safety-net hospital, it is essential to acknowledge several potential limitations. The patient population studied here was limited to the cardiology inpatient unit, so these results may not be representative of those with incident heart failure who may be diagnosed in the outpatient setting. Furthermore, this is a single-center retrospective study with a relatively small sample size. As a result, the statistical power and generalizability of the results from this study may be limited. Given the small sample size relative to the number of covariates, there is also the risk of overfitting in the multivariate regression model, impacting its reliability. As a retrospective study, there is also the potential for bias. Additionally, while we used traditional statistical techniques in this study, there is emerging research regarding the potential for machine learning to augment logistic regression and other statistical analyses in the prediction of cardiovascular disease [28,29]. In future studies, integrating machine learning with traditional statistical methods may provide enhanced predictive capabilities regarding complex interactions among study variables.

## 5. Conclusions

In this single-center, safety-net hospital study, we found that ischemic evaluation occurred in 50% of patients with newly diagnosed HF and identified that known CAD and higher SDI were significantly positively associated with ischemic workup in patients with newly diagnosed heart failure, while atrial fibrillation was significantly negatively associated with receiving ischemic workup. Notably, several demographic features, including age, sex, and race, as well as clinical features, including heart failure type, hypertension, hyperlipidemia, and diabetes, had no significant association with ischemic evaluation. The overall variation in ischemic evaluation trends observed in numerous studies necessitates standardization of testing with respect to current guidelines for patients with heart failure, given the mortality and quality of life benefits at stake. Our results add to the growing body of literature centered on better understanding variability in ischemic testing in patients with heart failure and compel further investigation to improve ischemic evaluation rates in this vulnerable patient population.

## Figures and Tables

**Table 1:** Baseline patient demographic and clinical characteristics reported as means ± SD or %.

Covariate		Overall (*n* = 200)	No Ischemic Evaluation (*n* = 101)	Ischemic Evaluation (*n* = 99)
Age		73.9 ± 16	79 ± 15.9	68.7 ± 14.4
Sex	Male	50	44.6	55.6
	Female	50	55.4	44.4
Race	White	51	63.4	38.4
	Black or African American	33.5	18.8	48.5
	Asian, Hawaiian, or Pacific Islander	5.5	8.9	2.0
	Other	10	8.9	11.1
Social Deprivation Index (SDI)		44.3 ± 31.9	33.5 ± 30.2	55.2 ± 30
HF Type	HF with preserved ejection fraction	37	41.6	32.3
	HF with reduced ejection fraction	41.5	37.6	45.4
	HF with mid-range ejection fraction	16	12.9	19.2
	HF with improved ejection fraction	1	0	2
NYHA HF Classification	Class II	1	2	0
	Class III	68	69	67
	Class IV	21	17	25
	Unknown	10	12	8
Pro-BNP		11,103 ± 16,711	9950 ± 12,689	12,243 ± 19,856
Coronary artery disease		35.5	30.7	40.4
Hypertension		87.5	82.2	92.9
Hyperlipidemia		30	22.8	37.4
Atrial fibrillation		36.5	49.5	23.2
Chronic kidney disease		35.5	31.7	39.4
Diabetes mellitus		42	34.7	49.5
Obesity		11.5	5.9	17.2

**Table 2:** Multivariate logistic regression model predicting the likelihood of an HF patient receiving ischemic evaluation.

Covariate		Odds Ratio	*p*-Value
Age		0.989	0.446
Sex	Male	Reference	Reference
	Female	0.858	0.723
Race	White	Reference	Reference
	Black or African American	1.960	0.159
	Asian, Hawaiian, or Pacific Islander	0.611	0.595
	Other	3.457	0.089
Social Deprivation Index (SDI)		1.022	0.003
HF Type	HF with preserved ejection fraction	Reference	Reference
	HF with reduced ejection fraction	0.521	0.171
	HF with mid-range ejection fraction	1.808	0.305
	HF with improved ejection fraction	3.72 × 10^8^	0.999
Coronary Artery Disease		2.816	0.015
Hypertension		2.082	0.200
Hyperlipidemia		1.647	0.238
Atrial fibrillation		0.240	0.001
Chronic kidney disease		1.404	0.394
Diabetes mellitus		1.932	0.089
Obesity		3.014	0.096

## Data Availability

Pertinent data are contained within the article.

## References

[1] Roger V.L. (2021). Epidemiology of Heart Failure. Circ. Res..

[2] Zheng J., Heidenreich P.A., Kohsaka S., Fearon W.F., Sandhu A.T. (2022). Variability in Coronary Artery Disease Testing for Patients With New-Onset Heart Failure. J. Am. Coll. Cardiol..

[3] Lala A., Desai A.S. (2014). The Role of Coronary Artery Disease in Heart Failure. Heart Fail. Clin..

[4] Martin S.S., Aday A.W., Almarzooq Z.I., Anderson C.A.M., Arora P., Avery C.L., Baker-Smith C.M., Barone Gibbs B., Beaton A.Z., Boehme A.K. (2024). 2024 Heart Disease and Stroke Statistics: A Report of US and Global Data From the American Heart Association. Circulation.

[5] Doshi D., Ben-Yehuda O., Bonafede M., Josephy N., Karmpaliotis D., Parikh M.A., Moses J.W., Stone G.W., Leon M.B., Schwartz A. (2016). Underutilization of Coronary Artery Disease Testing Among Patients Hospitalized With New-Onset Heart Failure. J. Am. Coll. Cardiol..

[6] O’Connor K.D., Brophy T., Fonarow G.C., Blankstein R., Swaminathan R.V., Xu H., Matsouaka R.A., Albert N.M., Velazquez E.J., Yancy C.W. (2020). Testing for Coronary Artery Disease in Older Patients With New-Onset Heart Failure: Findings From Get With The Guidelines–Heart Failure. Circ. Heart Fail..

[7] Tang P., Wang Q., Ouyang H., Yang S., Hua P. (2023). The Feasibility of Early Detecting Coronary Artery Disease Using Deep Learning-Based Algorithm Based on Electrocardiography. Aging.

[8] Velazquez E.J., Lee K.L., Jones R.H., Al-Khalidi H.R., Hill J.A., Panza J.A., Michler R.E., Bonow R.O., Doenst T., Petrie M.C. (2016). Coronary-Artery Bypass Surgery in Patients with Ischemic Cardiomyopathy. N. Engl. J. Med..

[9] Zheng J., Heidenreich P.A., Kohsaka S., Fearon W.F., Sandhu A.T. (2023). Long-Term Outcomes of Early Coronary Artery Disease Testing After New-Onset Heart Failure. Circ. Heart Fail..

[10] Robert Graham Center, American Academy of Family Physicians (2018). Social Deprivation Index. https://www.graham-center.org/maps-data-tools/social-deprivation-index.html.

[11] Gheorghiade M., Sopko G., De Luca L., Velazquez E.J., Parker J.D., Binkley P.F., Sadowski Z., Golba K.S., Prior D.L., Rouleau J.L. (2006). Navigating the Crossroads of Coronary Artery Disease and Heart Failure. Circulation.

[12] Smith S.C., Blair S.N., Bonow R.O., Brass L.M., Cerqueira M.D., Dracup K., Fuster V., Gotto A., Grundy S.M., Miller N.H. (2001). AHA/ACC Guidelines for Preventing Heart Attack and Death in Patients With Atherosclerotic Cardiovascular Disease: 2001 Update. Circulation.

[13] Maddox Thomas M., Januzzi James L., Allen Larry A., Khadijah B., Sara B., Javed B., Davis Leslie L., Fonarow Gregg C., Ibrahim Nasrien E., JoAnn L. (2024). 2024 ACC Expert Consensus Decision Pathway for Treatment of Heart Failure With Reduced Ejection Fraction. J. Am. Coll. Cardiol..

[14] Heidenreich P.A., Bozkurt B., Aguilar D., Allen L.A., Byun J.J., Colvin M.M., Deswal A., Drazner M.H., Dunlay S.M., Evers L.R. (2022). 2022 AHA/ACC/HFSA Guideline for the Management of Heart Failure: A Report of the American College of Cardiology/American Heart Association Joint Committee on Clinical Practice Guidelines. Circulation.

[15] Alghamry A., Hanna J., Pelecanos A., Kyranis S., Khelgi V., O’Rourke P., Carroll O., Oxenford C., Rangaswamaiah S., Tan C. (2016). Predictors of Significant Coronary Artery Disease in Atrial Fibrillation: Are Cardiac Troponins a Useful Measure. Int. J. Cardiol..

[16] Da Silva R.M.F.L. (2017). Influence of Inflammation and Atherosclerosis in Atrial Fibrillation. Curr. Atheroscler. Rep..

[17] Kassab K., Kattoor A.J., Doukky R. (2020). Ischemia and Viability Testing in New-Onset Heart Failure. Curr. Cardiol. Rep..

[18] Huang C.-W., Kohan S., Liu I.-L.A., Lee J.S., Baghdasaryan N.C., Park J.S., Vallejo J.D., Subject C.C., Nguyen H., Lee M.-S. (2024). Association Between Coronary Artery Disease Testing in Patients with New-Onset Heart Failure and Heart Failure Readmission and Mortality. J. Gen. Intern. Med..

[19] Severino P., Mancone M., D’Amato A., Mariani M.V., Prosperi S., Alunni Fegatelli D., Birtolo L.I., Angotti D., Milanese A., Cerrato E. (2024). Heart Failure ‘the Cancer of the Heart’: The Prognostic Role of the HLM Score. ESC Heart Fail..

[20] D’Amato A., Severino P., Mancone M., Mariani M.V., Prosperi S., Colombo L., Myftari V., Cestiè C., Labbro Francia A., Germanò R. (2024). Prognostic Assessment of HLM Score in Heart Failure Due to Ischemic Heart Disease: A Pilot Study. J. Clin. Med..

[21] Kimenai D.M., Pirondini L., Gregson J., Prieto D., Pocock S.J., Perel P., Hamilton T., Welsh P., Campbell A., Porteous D.J. (2022). Socioeconomic Deprivation: An Important, Largely Unrecognized Risk Factor in Primary Prevention of Cardiovascular Disease. Circulation.

[22] Vinter N., Fawzy A.M., Gent D., Ding W.Y., Johnsen S.P., Frost L., Trinquart L., Lip G.Y.H. (2022). Social Determinants of Health and Cardiovascular Outcomes in Patients with Heart Failure. Eur. J. Clin. Investig..

[23] Matetic A., Bharadwaj A., Mohamed M.O., Chugh Y., Chugh S., Minissian M., Amin A., Van Spall H., Fischman D.L., Savage M. (2020). Socioeconomic Status and Differences in the Management and Outcomes of 6.6 Million US Patients With Acute Myocardial Infarction. Am. J. Cardiol..

[24] Minkus H., Wenn P., Munshi R., Hai O., Makaryus A., Zeltser R. (2022). Abstract 223: Assessment of The Impact Of Social And Racial Determinants on Patients Admitted With Heart Failure to a Safety Net Hospital. Circ. Cardiovasc. Qual. Outcomes.

[25] Skelly A.C., Hashimoto R., Buckley D.I., Brodt E.D., Noelck N., Totten A.M., Lindner J.R., Fu R., McDonagh M. (2016). Introduction. Noninvasive Testing for Coronary Artery Disease [Internet].

[26] Parker M.W., Iskandar A., Limone B., Perugini A., Kim H., Jones C., Calamari B., Coleman C.I., Heller G.V. (2012). Diagnostic Accuracy of Cardiac Positron Emission Tomography Versus Single Photon Emission Computed Tomography for Coronary Artery Disease: A Bivariate Meta-Analysis. Circ. Cardiovasc. Imaging.

[27] Langdorf M.I., Wei E., Ghobadi A., Rudkin S.E., Lotfipour S. (2010). Echocardiography to Supplement Stress Electrocardiography in Emergency Department Chest Pain Patients. West. J. Emerg. Med..

[28] Srinivasan S., Gunasekaran S., Mathivanan S.K., M. B B.A.M., Jayagopal P., Dalu G.T. (2023). An Active Learning Machine Technique Based Prediction of Cardiovascular Heart Disease from UCI-Repository Database. Sci. Rep..

[29] Ogunpola A., Saeed F., Basurra S., Albarrak A.M., Qasem S.N. (2024). Machine Learning-Based Predictive Models for Detection of Cardiovascular Diseases. Diagnostics.

